# 

**Published:** 1818-09

**Authors:** 


### LECTURES.

*Adv.*]—Dr. Clutterbuck will begin his *Autumn* Course of Lectures on the *Theory and Practice of Physic, Materia Medica, and Chemistry*, on Friday, Oct. 2, at ten o'clock in the morning.—For particulars inquire at his house, No. 1, in the Crescent, New Bridge-street, Blackfriars; or at the General Dispensary, Aldersgate-street.

*Adv.*]—Mr. Guthrie will commence his Winter Course of Lectures on Surgery, on Monday, Oct. 5, at five minutes past eight in the evening, in the Waiting Room of the Royal Westminster Infirmary for Diseases of the Eye, Mary-le-bone-street, Piccadilly. To be continued on Mondays, Wednesdays, and Fridays. *Terms of attendance*—Perpetual, Five Guineas; Single Course, Three Guineas.

*Adv.*]—Dr. Davis will commence his Winter Lectures on the Theory and Practice of Midwifery, and on the Diseases of Women and Children; at his house, 29, George-street, Hanover-square, on Monday, the 5th of October, at half-past ten o'clock in the forenoon; and at Mr. Taunton's Theatre, Hatton-garden, on Tuesday, Oct. 6, at six o'clock in the evening.

*Adv.*]—Mr. Curtis, Aurist to H. R. H. the Prince Regent, and Surgeon to the Royal Dispensary for the Diseases of the Ear, will commence his next Course of Lectures on the Anatomy, Physiology, and Pathology, of the Ear, on Thursday, Oct. 1, at the Royal Dispensary, Carlisle-street.—For particulars apply to Mr. Curtis, at his own house, No. 2, Soho-square. A Clinical Lecture during the Course will be given on the most important Cases which occur at the Dispensary.

*Adv.*]—The Courses of Lectures will commence at St. George's Medical, Chemical, and Chirurgical, School, the first week of October next. —1. On the Laws of the Animal Economy, and the Practice of Physic, with occasional Pathological Demonstrations; by George Pearson, M.D. V.R.S. Senior Physician to St. George's Hospital, &c. &c.—2. On Chemistry, in which Medical Jurisprudence will be introduced as a Part of the Course; by W. T. Brande, Sec. R. S. Pr. of Chem. R. I.—3. On the Theory and Practice of Surgery; by B. C. Brodie, V. R. S. Assistant-Surgeon to St. George's Hospital, &c.—4. On Therapeutics, with Materia Medica; by George Pearson, M.D.—5. Surgical Lectures will be given gratuitously to the Pupils of St. George's Hospital by Sir E. Home, Bart.

Dr. George Gilbert Currey will commence his usual Course of Lectures on the Theory and Practice of Medicine and Materia Medica, on Friday, October 2.

Mr. Wilson and Mr. Bell will commence their Lectures on Anatomy, on the 1st of October.

An extended and practical Course of Lectures and Demonstrations on Chemistry will be delivered at the Royal Institution, by William Thomas Brande; to commence on the first Tuesday in October, at nine.

Dr. P. M. Latham and Dr. Southey will begin their Lectures upon the Practice of Physic and the Materia Medica, in the first week of October.

The following Courses of Lectures will be commenced at the Medical School, St. Bartholomew's-Hospital, on Thursday, October 1:—On the Theory and Practice of Medicine, by Dr. Hue—On Anatomy and Physiology, by Mr. Abernethy—On the Theory and Practice of Surgery, by Mr. Abernethy.—On Chemistry and Materia Medica, by Dr. Hue—On Midwifery, by Dr. Gooch—Practical Anatomy, by Mr. Stanley.

The Medical Lectures in the University of Glasgow will begin on Tuesday, Nov. 3, at the following hours:—Surgery, by Mr. Burns, at nine in the morning—Chemistry and Chemical Pharmacy, by Dr. Thomson, at ten—Midwifery, by Mr. Towers, at eleven—Practice of Medicine, by Dr. Freer, at twelve—Anatomy, by Dr. Jeffray, at two in the afternoon—Institutions of Medicine, by Dr. Freer, at three—Dietetics, Materia Medica, and Pharmacy, by Dr. Miller, at seven.

### BOOKS PREPARING FOR PUBLICATION.

Mr. STANLEY, Assistant-Surgeon and Demonstrator of Anatomy at St. Bartholomew's Hospital, is preparing for publication in October next, a Manual of Practical Anatomy, for the use of Students engaged in Dissections.

The Second Edition is in the Press, of the Elements of Conchology according to the Linnæan System; illustrated by twenty-eight Plates drawn from Nature. By the Rev. E. J. BURROW, A.M. F.R.S. F.L.S. Mem. Geol. Soc.

Dr. JAMES JOHNSON, Author of the Influence of Tropical Climates on European Constitutions, &c., will publish, on the 10th of the present month, the Influence of Civil Life, Sedentary Habits, and Intellectual Refinement, on Human Health and Human Happiness; including an Estimate of the Balance of Enjoyment and Suffering in the different Gradations of Society.

ANDERSON and CHASE, Medical Booksellers, 40, West-Smithfield, are preparing for publication, on the 1st of October next, their Annual Catalogue of New and Second-hand Medical Books; with a Complete List of the Lectures delivered in London, their Terms, Hours of Attendance, &c.

UNDERWOOD's Catalogue of Medical Books for 1818-19, with a List of the Lectures delivered in London, is in the press.

A New Edition of Dr. A. P. WILSON PHILIP's work on the Vital Functions will shortly appear.

In a few days will be published, in 8vo. an Inquiry into the Influence of Situation in Pulmonary Consumption, and on the Duration of Life; illustrated by Statistical Reports. By JOHN G. MANSFORD, Member of the Royal College of Surgeons of London.

In the press, Practical Instructions on the Treatment of Apparent Death, arising from Noxious Vapours, Drowning, Extreme Heat or Cold, Infants apparently Still-born, Persons poisoned; and on the Preventive Treatment of Persons bitten by Mad Dogs, &c. &c.; with Observations on these Accidents, and on the Signs which distinguish Actual Death from that which is only Apparent. Translated from the French of ANTOINE PORTAL, Professor of Medicine, Anatomy, &c. &c. &c.

---

### CATALOGUE OF MEDICAL BOOKS.

ANATOMICAL Examinations; a Complete Series of Anatomical Questions, with Answers: the Answers arranged so as to form an Elementary System of Anatomy, and intended as preparatory to Examination by the Royal College of Surgeons; to which are annexed Tables of the Bones, Muscles, and Arteries. 2 vols. 12mo. Fourth Edition.

A General Catalogue of Medical Books sold by Highley and Son; to which is added a Complete List of all the Lectures delivered in London, with the Terms, Hours of Attendance, &c.

A Succinct Account of the Contagious Fever of this Country, exemplified in the Epidemic now prevailing in London; with the appropriate Method of Treatment as practised in the House of Recovery. By Thos. Bateman, M.D. F.L.S. &c. 8vo. 6s.

Remarks on Burns and Scalds, chiefly in reference to the Principle of Treatment at the Time of their Infliction. By N. Dickinson, Esq. 8vo. 5s.

The Influence of the Atmosphere, more especially the Atmosphere of the British Isles, on the Health and Functions of the Human Frame; including Practical Observations on those derangements of the Liver, Digestive Organs, Heart, and Nervous System, resulting from Climatorial Influence, Irregularity of Living, Mental Anxiety, or Sedentary Habits. By James Johnson, Esq. 8vo. Second Edition. 9s.

A Treatise on the Virtues and Efficacy of a Crust of Bread ate early in a Morning, fasting; with some Critical Observations concerning the Recrements of the Blood. 8vo. Sixth Edition. 2s.

An Elementary Treatise on Mineralogy and Geology, being an Introduction to the Study of these Sciences, and designed for the use of Pupils, for Persons attending Lectures on these Subjects, and as a Companion for Travellers in the United States of America; illustrated by Six Plates. By Parker Cleveland, Professor of Mathematics, &c. 8vo. 1l. 1s.

A Plan of the Fœtal Circulation; with References. By W. Horner, M.D. 5s.

---

*Books lately imported by Burgess and Hill.*

Alibert Descriptions des Maladies de la Peau; avec belle grav. color. 10 Livraisons. Folio, Proof Impressions of the Plates.

Scarpa (Anto.) Saggio di Operasioni e d'Esperienze sulle principali Malettie degli Occhi; with a beautiful Plate of the Author.

Traité des Maladies des Yeux; avec des Planches coloriers representant ces Maladies d'après Nature, suivi de la Description de l'Œil Humain, traduite du Latin de S. T. Soemmering. Par A. P. Demours. 4 vols.

Walteri (J. G.) Observationes Anatomiæ. Folio.

Tabulæ Anatomico Pathologicæ Modos omnes, quibus Partium Corporis Humani omnium Forma externa atque Interna a norma recedit; exhibentes Auctore J. F. Meckel. Folio.

Icones ad Illustrandam Anatomen Comparatum. Folio, Plates.

De Duplicitate Monstrosa Commentarius quem Conscripsit J. F. Mickel. Folio, Plates.

Traité Pratique des Hernies. Par Antoine Scarpa. Fig.

Reflexions et Observations Anatomico Chirurgicales sur l'Aneurisme. Par A. Scarpa. Large Folio.

---

### *TO CORRESPONDENTS.*

*It is with much regret that we inform* Mr. W. *that his last communication cannot be inserted in our Journal. In explanation of which, we have only to repeat what we have frequently stated; that controversies not tending to benefit the public, or increase our stock of useful knowledge, and which are better adapted for the arbitration of the friends of the parties, or a court of justice, cannot be allowed to occupy those pages we are so anxious to devote to the reception of useful and interesting matter.*

"Supplex" *is informed that we cannot trace any immediate connexion between what he considers as cause and effect. The particular act to which he alludes, was certainly not an improper one, and more probably averted than produced ill consequences.*

Mr. S. *will find that twenty grains of carbonate of soda, and twenty-five of tartaric acid, dissolved separately, and then mixed, will make good effervescing soda water. The above, with a proportion of sugar mixed with the carbonate of soda, are what are usually sold as sodaic powders.*

*The Proprietors of this Journal have the satisfaction of announcing to their Readers, that they have recently engaged additional Editors to this Journal, and have made extensive arrangements with some of the most eminent men of the profession in America, France, and Germany; whereby they will be enabled to enrich their pages every month with accurate accounts of every thing that is new and valuable in Foreign Medicine and Surgery. They also take this opportunity to inform their Subscribers, that the* General Index *to the first* Thirty-four *Volumes of the Journal is now in great forwardness, and, they have no doubt, will be ready for delivery by Christmas. As very few Copies more than are previously subscribed for will be printed, it is requested that such Gentlemen as wish to secure a Copy, and have not already sent in their Names, will do so without loss of time, in order to prevent disappointment.*

*Several Communications have arrived, all of which shall appear in our next number, if possible.*

